# Supplementary material for: Genetic diversity of 1,845 rhesus macaques improves genetic variation interpretation and identifies disease models
Source: Nat Commun. 2024 Jul 5;15:5658. doi: 10.1038/s41467-024-49922-6 (PMC11226599; doi:10.1038/s41467-024-49922-6)
Supplement: Supplementary file 1 — Supplementary Information [file 41467_2024_49922_MOESM1_ESM.pdf]

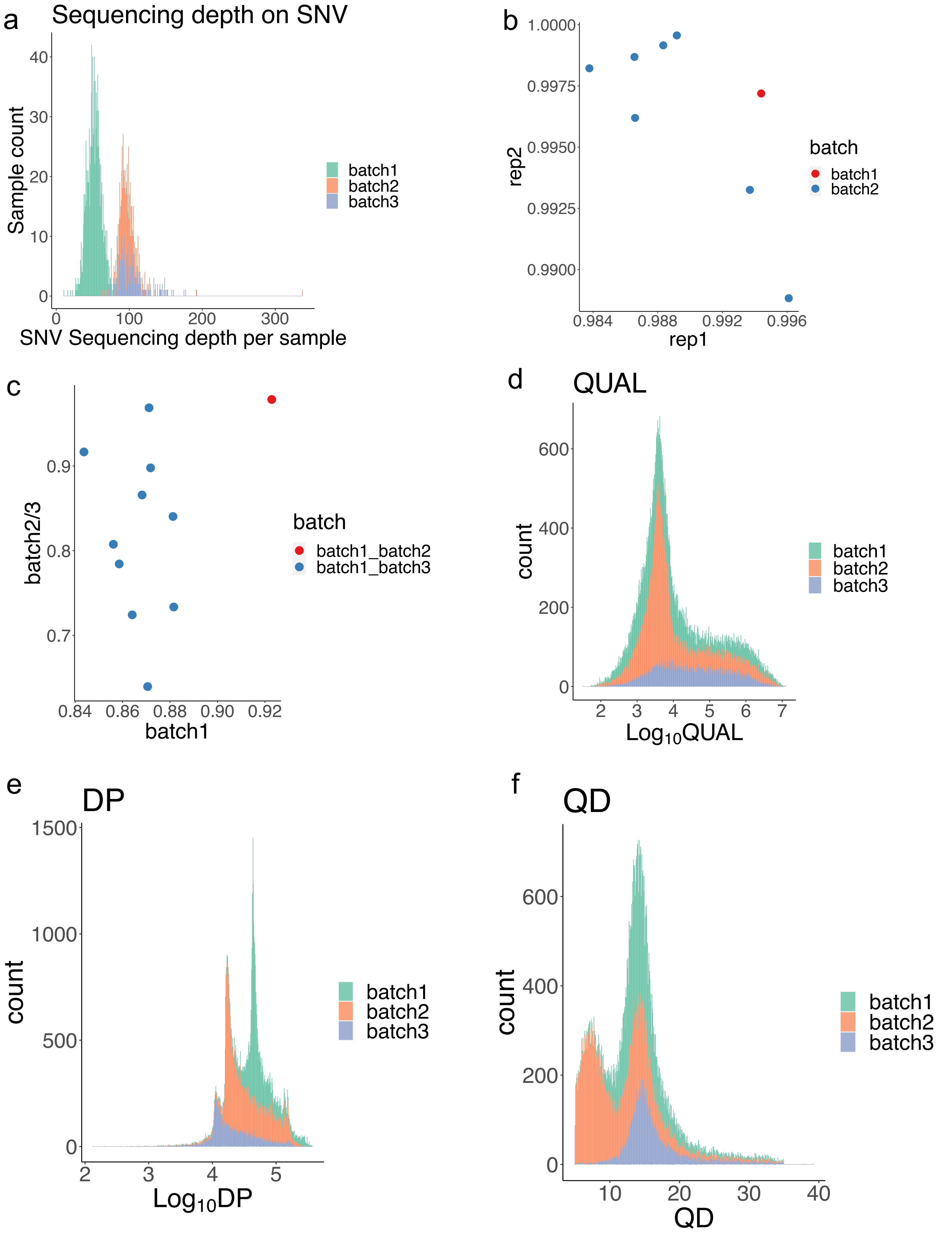

a
Sequencing depth on SNV
Sample count
SNV Sequencing depth per sample
0
10
20
30
40
0
100
200
300
batch1
batch2
batch3
b
rep2
rep1
1.0000
0.9975
0.9950
0.9925
0.9900
0.984
0.988
0.992
0.996
batch
batch1
batch2
c
batch2/3
batch1
0.9
0.8
0.7
0.84
0.86
0.88
0.90
0.92
batch
batch1_batch2
batch1_batch3
d
QUAL
count
Log10QUAL
0
200
400
600
2
3
4
5
6
7
batch1
batch2
batch3
e
DP
count
Log10DP
0
500
1000
1500
2
3
4
5
batch1
batch2
batch3
f
QD
count
QD
0
200
400
600
10
20
30
40
batch1
batch2
batch3

Supplementary Fig. 1: QC metrics of next generation sequencing

a, A histogram plot showing the sequencing read coverage at SNVs sites, with color indicating the primary experiment batches of targeted-sequencing. All the samples have higher than 10x sequencing coverage. **b,** A scatter plot showing the overlapped SNVs proportions of two technical replicates within the same batch from 8 animals, with color indicating the primary batch. X axis indicates the shared SNVs proportion in replicate 1. Y axis indicates the shared SNVs proportion in replicate 2. **c,** A scatter plot showing the overlapped SNVs proportions of two technical replicates from 11 animals sequenced in two different batches, with color indicating the primary batch pairs. X axis indicates the shared SNVs proportion in replicate 1 from batch1. Y axis indicates the shared SNVs proportion in replicate 2 from batch2 or batch3. **d,** A histogram plot showing the distribution of $\log_{10}$QUAL score (variant quality score) from GATK, with color indicating the primary experiment batches. After QC, all SNVs have QUAL $\geq$30. **e**, A histogram plot showing the distribution of $\log_{10}$ DP (Approximate read depth) from GATK, with color indicating the primary experiment batches. **f**, A histogram plot showing the distribution of QD (the QUAL score normalized by allele depth (AD) for a variant) from GATK, with color indicating the primary experiment batches. After QC, all SNVs have QD ≥ 5. Source data are provided in the Source Data file. The QC matrix in d,e and f covers 59,256 genetic variants.

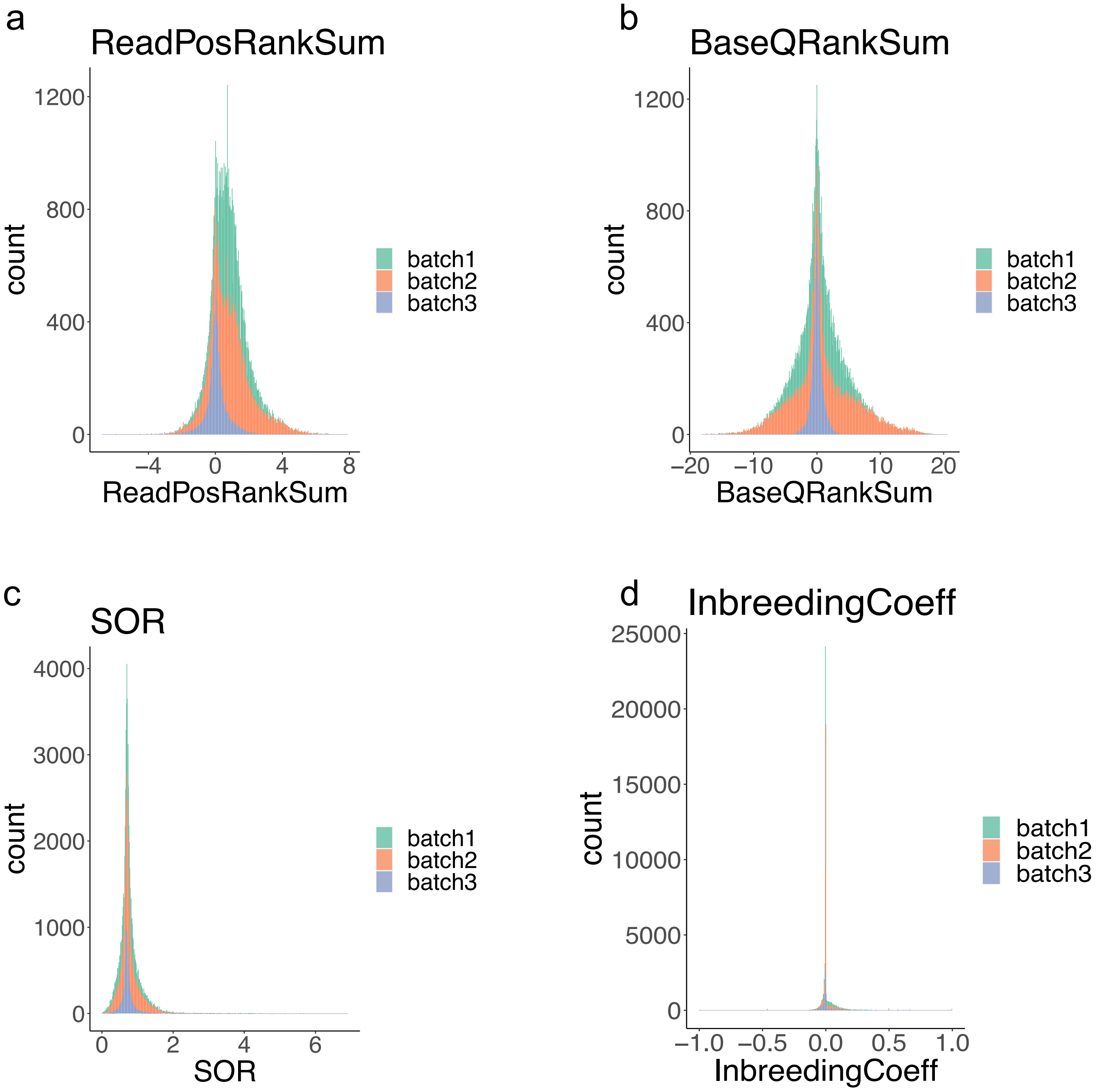

Supplementary Fig. 2: Additional QC metrics of next generation sequencing
a, A histogram plot showing the distribution of ReadPosRankSum (relative positioning of REF versus ALT alleles within reads, the ideal value is close to 0) from GATK, with color indicating the primary experiment batches. After QC, all SNVs have ReadPosRankSum ≥ -8. **b**, A histogram plot showing the distribution of BaseQRankSum (compares the base qualities of the data supporting the reference allele with those supporting the alternate allele. The ideal value is close to zero) from GATK, with color indicating the primary experiment batches. **c**, A histogram plot showing the distribution of SOR (Allele-specific strand bias estimated by the Symmetric Odds Ratio test) from GATK, with color indicating the primary experiment batches. **d**, A histogram plot showing the distribution of Inbreeding Coefficient (measures the excess heterozygosity at a variant site) from GATK, with color indicating the primary experiment batches. Source data are provided in the Source Data file. The QC matrix in a, b, c and d covers 59,256 genetic variants.

a 36444:RPGRIP1:NM_020366:exon13:c.T1736A:p.L579X

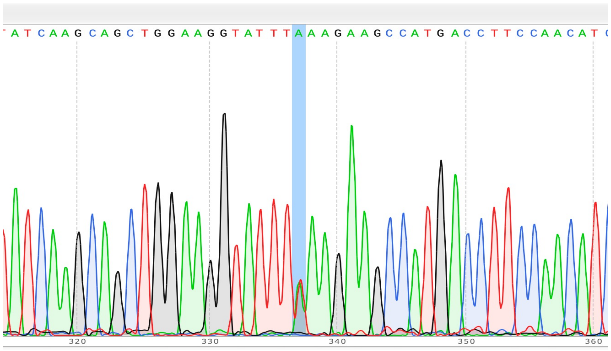

b 36451:*PLA2G6*:NM_003560:c.1078-2A>G

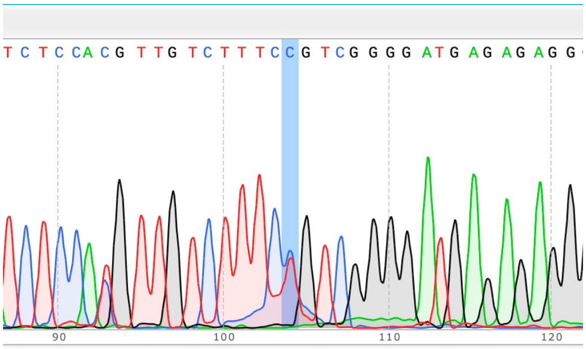

c 105483:EYS:NM_001292009:c.C8608T:p.R2870X

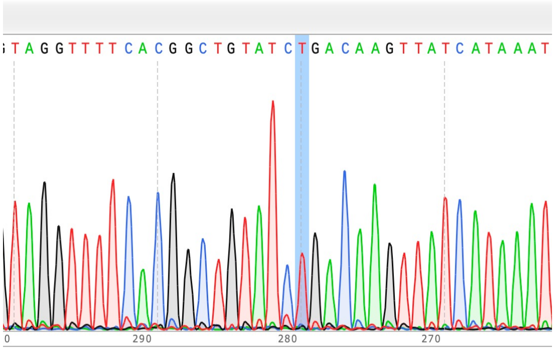

d 35821:ABCA4:NM_000350:c.G6416A:p.R2139Q

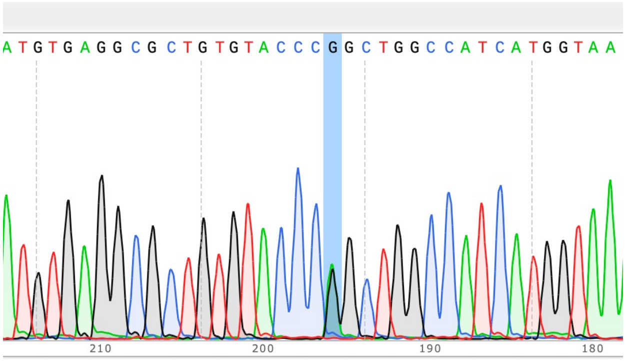

e 36441:ABCA4:NM_000350:c.G6416A:p.R2139Q

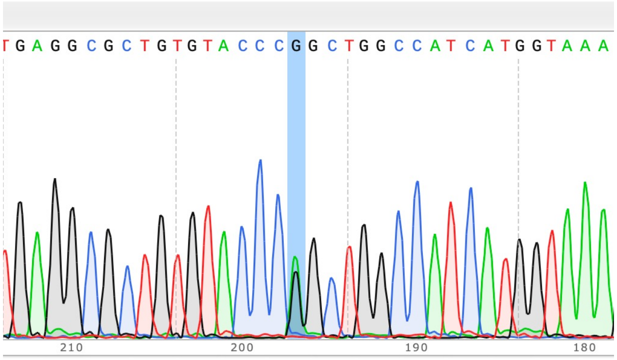

Supplementary Fig. 3: Sanger validation of putative pathogenic variants

The allele locations were highlighted in blue. **a**, Sanger result showing a rhesus macaque (36444) carrying a heterozygous splice acceptor variant (NM_020366:exon13:c.T1736A:p.L579X) in *RPGRIP1*. **b,** Sanger result showing a rhesus macaque (36451) carrying a heterozygous splice acceptor variant (NM_003560:c.1078-2A>G) in *PLA2G6*. **c,** Sanger result showing a rhesus macaque (105483) carrying a homozygous stop-gained variant (NM_001292009:c.C8608T:p.R2870X) in *EYS*. **d, e** Sanger result showing two rhesus macaques (35821 and 36441) from two colonies, carrying a heterozygous missense variant (NM_000350:c.G6416A:p.R2139Q) in *ABCA4*.

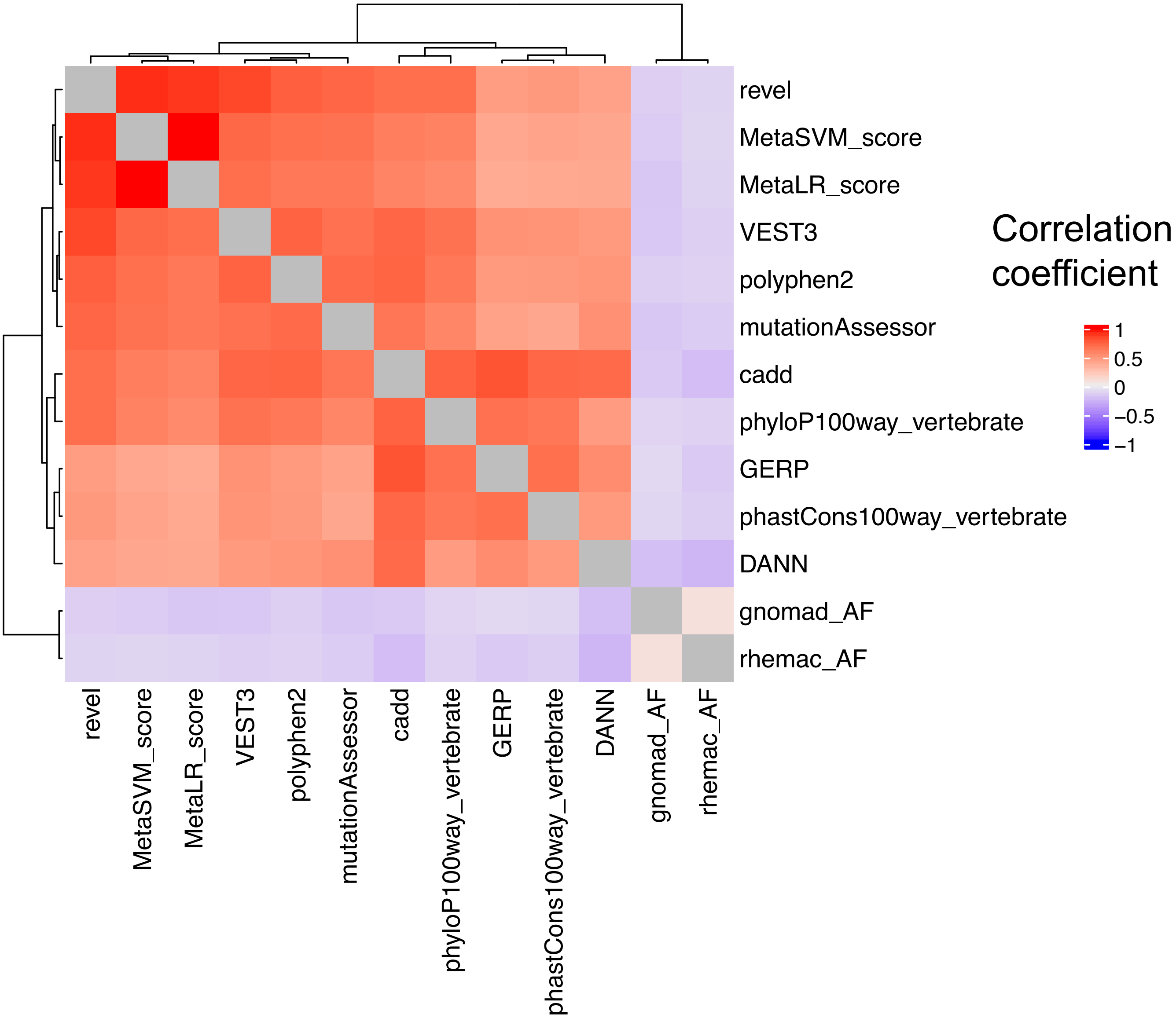

Supplementary Fig. 4: Correlation between the 13 features incorporated into the machine-learning model

The heatmap showing the correlation between the 13 features incorporated into the machine-learning model to develop an integrative score for predicting the pathogenicity of missense variants. This correlation is calculated based on the 13 scores of 10836 missense variants. The diagonal was grey out as it represents self-comparison, which are not informative. Source data are provided in the Source Data file.

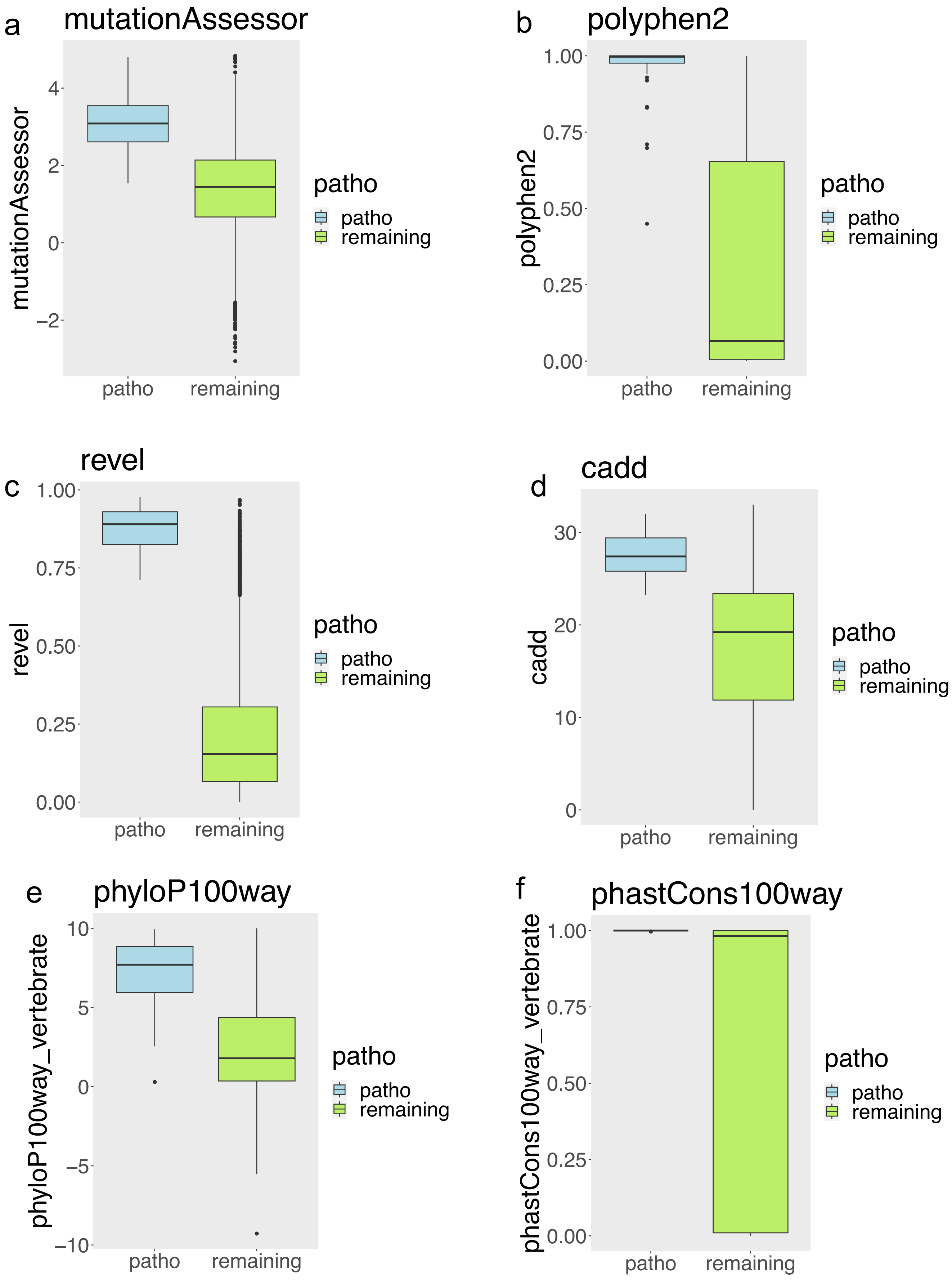
a
mutationAssessor
mutationAssessor
4
2
0
−2
patho
remaining
patho
patho
remaining
b
polyphen2
polyphen2
1.00
0.75
0.50
0.25
0.00
patho
remaining
patho
patho
remaining
c
revel
revel
1.00
0.75
0.50
0.25
0.00
patho
remaining
patho
patho
remaining
d
cadd
cadd
30
20
10
0
patho
remaining
patho
patho
remaining
e
phyloP100way
phyloP100way_vertebrate
10
5
0
−5
−10
patho
remaining
patho
patho
remaining
f
phastCons100way
phastCons100way_vertebrate
1.00
0.75
0.50
0.25
0.00
patho
remaining
patho
patho
remaining

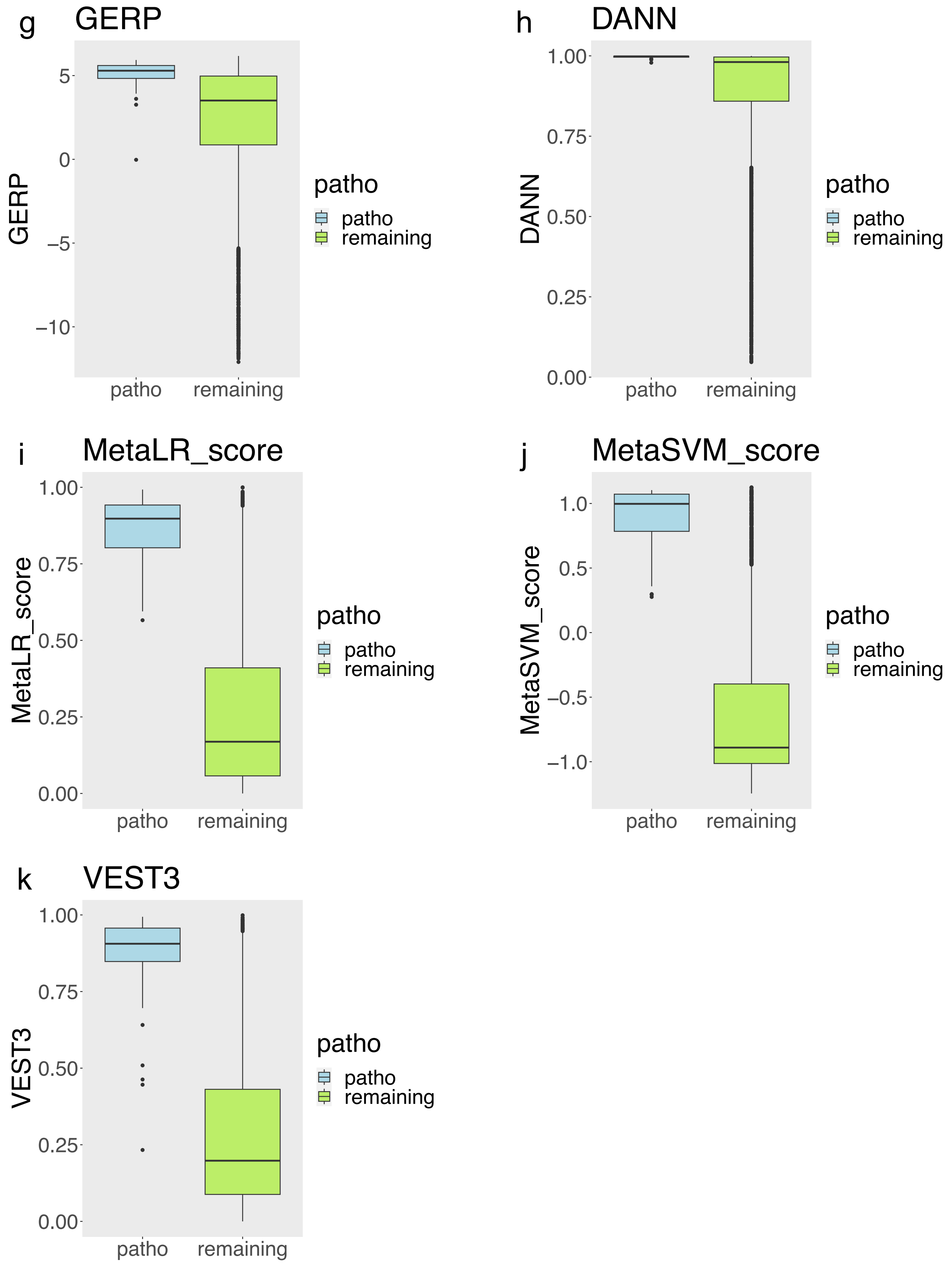

g
GERP
5
0
−5
−10
GERP
patho
remaining
patho
patho
remaining
h
DANN
1.00
0.75
0.50
0.25
0.00
DANN
patho
remaining
patho
patho
remaining
i
MetaLR_score
1.00
0.75
0.50
0.25
0.00
MetaLR_score
patho
remaining
patho
patho
remaining
j
MetaSVM_score
1.0
0.5
0.0
−0.5
−1.0
MetaSVM_score
patho
remaining
patho
patho
remaining
k
VEST3
1.00
0.75
0.50
0.25
0.00
VEST3
patho
remaining
patho
patho
remaining

Supplementary Fig. 5: The distribution of established variant prediction scores of the 6,195 rhesus macaque variants

The variants predicted as likely pathogenic by HM score (n=61) are colored in blue. The remaining variants (n=6134) are colored in green. Box plot showing the distribution of a) mutationAssessor score, b) polyphen2 score, c) revel score, d) CADD score, e) phyloP100way score, f) phastCons100way score, g) GERP score, h) DANN score, i) MetaLR score, j) MetaSVM score, k) VEST3 score. The center of a box represents the median, while the lower and upper bounds correspond to the first and third quartiles, respectively. The whiskers extend up to 1.5 times the interquartile range, and the minima and maxima represent the observed minimum and maximum values. Source data are provided in the Source Data file.

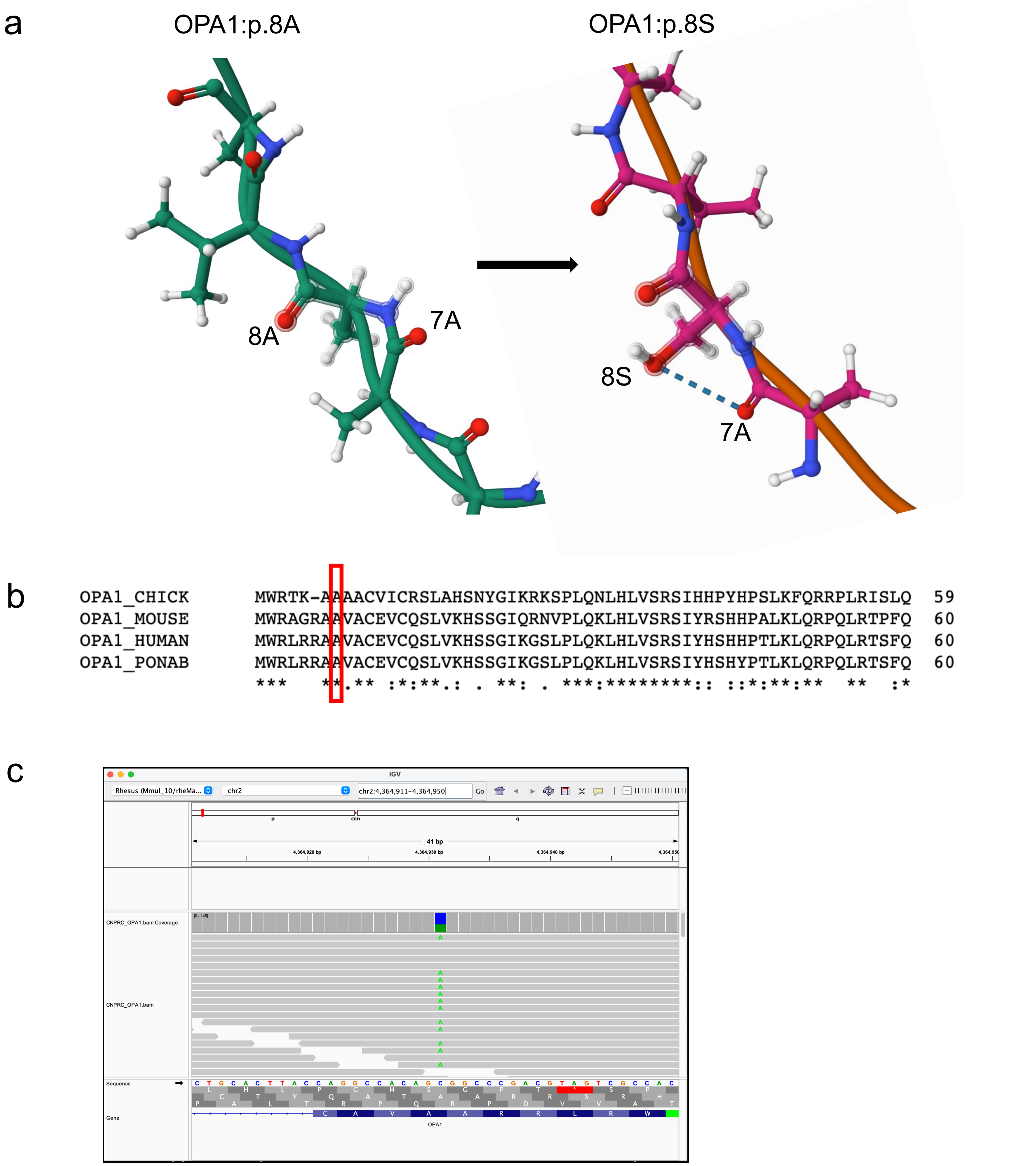

Supplementary Fig. 6: A putative pathogenic variant, OPA1:p.A8S, associated with autosomal-dominant optic atrophy

a, The protein structure of OPA1 carrying the reference allele (p.8A, left) and putative pathogenic allele (p.8S, right) simulated by AlphaFold. A hydrogen bond forms between p.7A and p.8S of OPA1, which is absent in the reference copy of OPA1 between p.7A and p.8A. This result suggests this variant may impact the mitochondrial targeting signal region of the OPA1 protein. b, The sequence alignment showing OPA1:p.8A is conserved across vertebrates. **c,** The IGV result of the OPA1:p.A8S in the 28-year-old rhesus macaque with the most severe phenotype.

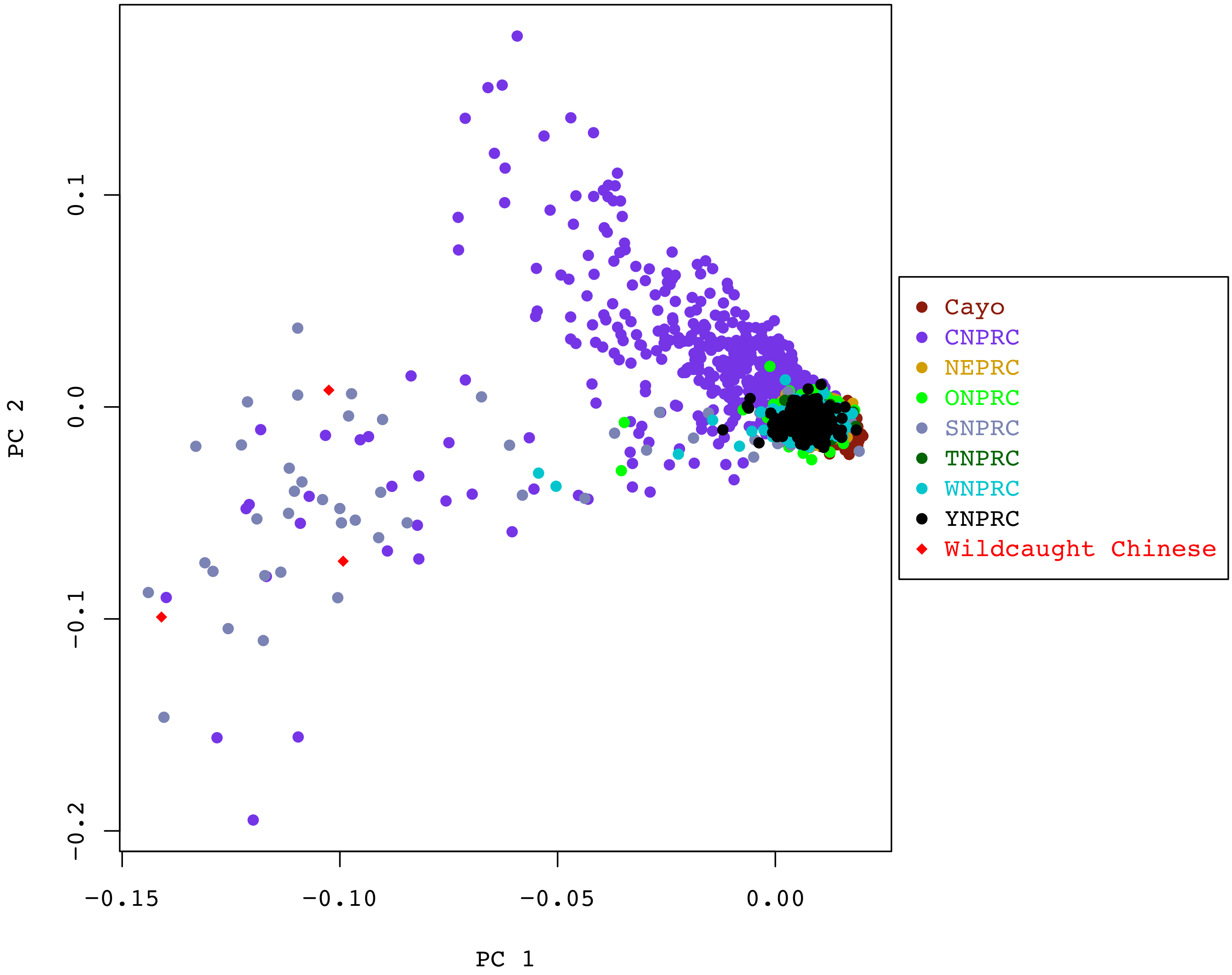

Supplementary Fig. 7: Principal component analysis (PCA) for the origins of the rhesus macaque populations

This PCA plot is based on the genotype data of 1845 rhesus macaques in this study along with the genotype data of 3 wild-caught Chinese rhesus macaques. Source data are provided in the Source Data file.
