# Supplementary material for: Genetic diversity of 1,845 rhesus macaques improves genetic variation interpretation and identifies disease models
Source: Nat Commun. 2024 Jul 5;15:5658. doi: 10.1038/s41467-024-49922-6 (PMC11226599; doi:10.1038/s41467-024-49922-6)
Supplement: Supplementary file 3 — Description of Additional Supplementary Files [file 41467_2024_49922_MOESM3_ESM.pdf]

Description of Additional Supplementary Files

Supplementary Data 1

Description: The list of primate research centers and number of animals sequenced.

Supplementary Data 2:

Description: The list of 374 targeted genes.

Supplementary Data 3:

Description: The 43 sequenced loss of function variants according to gene annotation in both macaques and humans.

Supplementary Data 4:

Description: The 31 reported putative human pathogenic variants.

Supplementary Data 5:

Description: The 19,417 filtered putative benign variants based on allele frequency in macaque and human populations.

Supplementary Data 6:

Description: The HM score of the 6,195 identified missense variants.

Supplementary Data 7:

Description: The age and sex information of the 8 OPA1 mutant rhesus macaques and 8 age-, sex-matched rhesus macaque controls.

Supplementary Data 8:

The primers used to validate putative pathogenic variants.
