# Supplementary material for: Genetic diversity of 1,845 rhesus macaques improves genetic variation interpretation and identifies disease models
Source: Nat Commun. 2024 Jul 5;15:5658. doi: 10.1038/s41467-024-49922-6 (PMC11226599; doi:10.1038/s41467-024-49922-6)
Supplement: Supplementary file 12 — Reporting Summary [file 41467_2024_49922_MOESM12_ESM.pdf]

nature portfolio

Corresponding author(s): Rui Chen

Last updated by author(s): Dec 16, 2023

Reporting Summary

Nature Portfolio wishes to improve the reproducibility of the work that we publish. This form provides structure for consistency and transparency in reporting. For further information on Nature Portfolio policies, see our Editorial Policies and the Editorial Policy Checklist.

Statistics

For all statistical analyses, confirm that the following items are present in the figure legend, table legend, main text, or Methods section.

n/a	Confirmed	
☐	☒	The exact sample size (*n*) for each experimental group/condition, given as a discrete number and unit of measurement
☐	☒	A statement on whether measurements were taken from distinct samples or whether the same sample was measured repeatedly
☐	☒	The statistical test(s) used AND whether they are one- or two-sided<br>*Only common tests should be described solely by name; describe more complex techniques in the Methods section.*
☒	☐	A description of all covariates tested
☒	☐	A description of any assumptions or corrections, such as tests of normality and adjustment for multiple comparisons
☐	☒	A full description of the statistical parameters including central tendency (e.g. means) or other basic estimates (e.g. regression coefficient) AND variation (e.g. standard deviation) or associated estimates of uncertainty (e.g. confidence intervals)
☐	☒	For null hypothesis testing, the test statistic (e.g. *F*, *t*, *r*) with confidence intervals, effect sizes, degrees of freedom and *P* value noted<br>*Give P values as exact values whenever suitable.*
☒	☐	For Bayesian analysis, information on the choice of priors and Markov chain Monte Carlo settings
☒	☐	For hierarchical and complex designs, identification of the appropriate level for tests and full reporting of outcomes
☒	☐	Estimates of effect sizes (e.g. Cohen's *d*, Pearson's *r*), indicating how they were calculated

Our web collection on statistics for biologists contains articles on many of the points above.

Software and code

Policy information about availability of computer code

Data collection	The "Genome sequencing" section in the Methods part details the experimental process to obtain the genomic sequencing data. The DNA samples were pooled for Agilent SureSelect Target enrichment system following manufacturer's protocol with modification. The library was sequenced in an Illumina Novaseq 6000 Sequencer to obtain the sequencing reads.
Data analysis	The code used for this project has been made public in the GitHub repository: https://github.com/fe4960/rhemac_retcap. A copy of the code version used for this publication is available from Zenodo (https://doi.org/10.5281/zenodo.11166726). There is also a "code availability" section specified in the manuscript.<br>Softwares used:<br>R 4.0.0<br>PLINK v1.90b5.2<br>BWA mem (0.7.12-r1039)<br>GATK 3.3-0-g37228af<br>GATK 4.0.0.0<br>liftOver from UCSC genome browser<br>bcftools 1.17<br>macse_v0.9b1.jar<br>paml4.9j<br>ensembl-vep v.101<br>ANNOVAR v. 07/17/2017<br>dbNSFP v.3.5a<br>HGMD (v.12-20-2016)

ClinVar (v20230710)
The gnomAD database (v2.1.1)
CADD (v1.6)
caret_6.0-93
cvAUC_1.1.4
JQuery version 2.1.4
MySQL 8.0.20
Apache 2.4.6
Bootstrap version 3.1.1
igv.js (2.15.11)

For manuscripts utilizing custom algorithms or software that are central to the research but not yet described in published literature, software must be made available to editors and reviewers. We strongly encourage code deposition in a community repository (e.g. GitHub). See the Nature Portfolio guidelines for submitting code & software for further information.

Data

Policy information about availability of data

All manuscripts must include a data availability statement. This statement should provide the following information, where applicable:

- Accession codes, unique identifiers, or web links for publicly available datasets
- A description of any restrictions on data availability
- For clinical datasets or third party data, please ensure that the statement adheres to our policy

A "Data availability" statement is specified in the manuscript as the following:
The rhesus macaque genome assembly Mmul_8.0.1 [https://www.ncbi.nlm.nih.gov/datasets/genome/GCF_000772875.2/] and Mmul_10 [https://www.ncbi.nlm.nih.gov/datasets/genome/GCF_003339765.1/] used in this study are available from the NCBI. The human genome assembly hg19 [https://hgdownload.soe.ucsc.edu/goldenPath/hg19/bigZips/hg19.fa.gz] used in this study is available in UCSC genome browser. The genome sequences of three wild caught Chinese rhesus samples used in this study are available in the Sequence Read Archive (SRA) under the sample accession numbers: SAMN03264758 [https://www.ncbi.nlm.nih.gov/biosample/SAMN03264758/], SAMN03264759 [https://www.ncbi.nlm.nih.gov/biosample/SAMN03264759/] and SAMN03264760 [https://www.ncbi.nlm.nih.gov/biosample/SAMN03264760/] 41. The mCED browser is accessible at https://ird.research.bcm.edu/macaque/, or https://rchenlab.github.io/resources/, The sequencing data generated in this study (i.e. bam files) have been deposited in the SRA under the accession number PRJNA1107273 [https://www.ncbi.nlm.nih.gov/bioproject/PRJNA1107273]. Source data are provided with this paper.

Research involving human participants, their data, or biological material

Policy information about studies with human participants or human data. See also policy information about sex, gender (identity/presentation), and sexual orientation and race, ethnicity and racism.

Reporting on sex and gender	N/A
Reporting on race, ethnicity, or other socially relevant groupings	N/A
Population characteristics	N/A
Recruitment	N/A
Ethics oversight	N/A

Note that full information on the approval of the study protocol must also be provided in the manuscript.

Field-specific reporting

Please select the one below that is the best fit for your research. If you are not sure, read the appropriate sections before making your selection.

☒ Life sciences ☐ Behavioural & social sciences ☐ Ecological, evolutionary & environmental sciences

For a reference copy of the document with all sections, see nature.com/documents/nr-reporting-summary-flat.pdf

Life sciences study design

All studies must disclose on these points even when the disclosure is negative.

Sample size	No statistical method was used to predetermine sample size. Given the low prevalence of the inherited retinal and neurodevelopment diseases, a substantial cohort of individuals should be screened. Assuming the aggregated prevalence of these diseases is 1 in 1000 and if under a recessive inheritance mode, in every 50 individuals, there should be 3 pathogenic alleles. Therefore, we sequenced 1,845 rhesus macaques across eight primate research centers in the United States. Supplementary Table S1 lists the number of the rhesus macaques collected from each primate research center.

Data exclusions	After quality control, the samples with genomic sequencing coverage < 10x and with no variants left after variant filtering were excluded. The quality control procedure is listed in the "Quality Control" section in the Methods part.
Replication	We repeated genomic sequencing on the samples from the same rhesus macaques to check data quality. This analysis can be found in the Supplementary Figure S1b and S1c.
Randomization	There is no randomization. For phenotyping analyses of the OPA1 heterozygous rhesus macaques, we collected OPA1 heterozygous rhesus macaques and age-, sex- matched OPA1 wild type rhesus macaques to control covariates.
Blinding	The Investigators were not blinded to allocation during experiments and outcome assessment. For phenotyping analyses of the OPA1 heterozygous rhesus macaques, the researchers knew genotype when they conducted manual measurements. However, automated measurements were generated by the software.

Reporting for specific materials, systems and methods

We require information from authors about some types of materials, experimental systems and methods used in many studies. Here, indicate whether each material, system or method listed is relevant to your study. If you are not sure if a list item applies to your research, read the appropriate section before selecting a response.

Materials & experimental systems

n/a	Involved in the study
☒	☐ Antibodies
☒	☐ Eukaryotic cell lines
☒	☐ Palaeontology and archaeology
☐	☒ Animals and other organisms
☒	☐ Clinical data
☒	☐ Dual use research of concern
☒	☐ Plants

Methods

n/a	Involved in the study
☒	☐ ChIP-seq
☒	☐ Flow cytometry
☒	☐ MRI-based neuroimaging

Animals and other research organisms

Policy information about studies involving animals; ARRIVE guidelines recommended for reporting animal research, and Sex and Gender in Research

Laboratory animals	All animals included in this study were adult rhesus macaques (Macaca mulatta) born and maintained at the eight primate research colonies listed in Supplementary Table S1.
Wild animals	No wild animals were used in the study.
Reporting on sex	We sequenced 1,845 animals regardless of sex. Since sex information were not necessary for sequencing and the overall study, we did not collect this data. However, for the phenotypic study of the rhesus macaque OPA1 model, we provided detailed sex information for the animals that underwent phenotyping in the Supplementary Data 7.
Field-collected samples	No field collected samples were used in the study.
Ethics oversight	All collected animals are accredited by the Association for Assessment and Accreditation of Laboratory Animal Care (AAALAC) International. This study was conducted in accordance with the Guidelines of the Association for Research in Vision and Ophthalmology Statement for the Use of Animals in Ophthalmic and Vision Research and the National Institutes of Health (NIH) Guide for the Care and Use of Laboratory Animals. Phenotyping and ophthalmic examinations were performed according to an animal protocol approved by the UC Davis Institutional Animal Care and Use Committee.

Note that full information on the approval of the study protocol must also be provided in the manuscript.

Plants

Seed stocks	*Report on the source of all seed stocks or other plant material used. If applicable, state the seed stock centre and catalogue number. If plant specimens were collected from the field, describe the collection location, date and sampling procedures.*
Novel plant genotypes	*Describe the methods by which all novel plant genotypes were produced. This includes those generated by transgenic approaches, gene editing, chemical/radiation-based mutagenesis and hybridization. For transgenic lines, describe the transformation method, the number of independent lines analyzed and the generation upon which experiments were performed. For gene-edited lines, describe the editor used, the endogenous sequence targeted for editing, the targeting guide RNA sequence (if applicable) and how the editor was applied.*
Authentication	*Describe any authentication procedures for each seed stock used or novel genotype generated. Describe any experiments used to assess the effect of a mutation and, where applicable, how potential secondary effects (e.g. second site T-DNA insertions, mosiacism, off-target gene editing) were examined.*
